# High opsonic phagocytosis activity and growth inhibition of merozoites are associated with RON4 antibody levels and protect against febrile malaria in Ghanaian children

**DOI:** 10.3389/fimmu.2023.1161301

**Published:** 2023-05-01

**Authors:** Eric Kyei-Baafour, Kwadwo Asamoah Kusi, Fareed K.N. Arthur, Regis W. Tiendrebeogo, Eunice Owusu-Yeboa, Susheel K. Singh, Sarah Friedrich, Thomas A. Gerds, Daniel Dodoo, Michael Theisen, Bright Adu

**Affiliations:** ^1^Department of Immunology, Noguchi Memorial Institute for Medical Research, College of Health Sciences, University of Ghana, Legon Accra, Ghana; ^2^Department of Biochemistry and Biotechnology, College of Science, Kwame Nkrumah University of Science and Technology, Kumasi, Ghana; ^3^Centre for Medical Parasitology at Department of International Health, Immunology and Microbiology, University of Copenhagen and Department of Infectious Diseases, Copenhagen University Hospital, Rigshospitalet, Copenhagen, Denmark; ^4^Department for Congenital Disorders, Statens Serum Institut, Copenhagen, Denmark; ^5^Section of Biostatistics, Department of Public Health, University of Copenhagen, Copenhagen, Denmark; ^6^Department of Medical Statistics, University Medical Center Goettingen, Goettingen, Germany

**Keywords:** malaria, opsonic phagocytosis, growth inhibition, merozoite protein, ELISA

## Abstract

**Background:**

Naturally acquired immunity to malaria may involve different immune mechanisms working in concert, however, their respective contributions and potential antigenic targets have not been clearly established. Here, we assessed the roles of opsonic phagocytosis and antibody-mediated merozoite growth inhibition in *Plasmodium falciparum (P. falciparum)* infection outcomes in Ghanaian children.

**Methods:**

The levels of merozoite opsonic phagocytosis, growth inhibition activities and six *P. falciparum* antigen-specific IgG of plasma samples from children (n=238, aged 0.5 to 13 years) were measured at baseline prior to the malaria seasons in southern Ghana. The children were then actively and passively followed up for febrile malaria and asymptomatic *P. falciparum* infection detection in a 50-week longitudinal cohort. *P. falciparum* infection outcome was modelled as a function of the measured immune parameters while accounting for important demographic factors.

**Results:**

High plasma activity of opsonic phagocytosis [adjusted odds ratio (aOR)= 0.16; 95%CI= 0.05 - 0.50, p = 0.002], and growth inhibition (aOR=0.15; 95% CI = 0.04-0.47; p = 0.001) were individually associated with protection against febrile malaria. There was no evidence of correlation (b= 0.13; 95% CI= -0.04-0.30; p=0.14) between the two assays. IgG antibodies against MSPDBL1 correlated with opsonic phagocytosis (OP) while IgG against *Pf*Rh2a correlated with growth inhibition. Notably, IgG antibodies against RON4 correlated with both assays.

**Conclusion:**

Opsonic phagocytosis and growth inhibition are protective immune mechanisms against malaria that may be acting independently to confer overall protection. Vaccines incorporating RON4 may benefit from both immune mechanisms.

## Introduction

Mechanisms of naturally acquired immune protection against *Plasmodium falciparum* febrile malaria, albeit not completely understood, may collectively account for several averted clinical malaria episodes in endemic populations. It has been established that naturally acquired immunity (NAI) against malaria is non-sterile and depends on persistent low-level parasitaemia and develops after repeated exposure to different parasite variants (1). The role of antibodies in NAI against malaria was demonstrated by passive immunoglobulin (IgG) transfer experiments (2, 3). Protective antibodies target antigens from different stages of the parasite including merozoites (4, 5). However, the underlying immune mechanisms and the critical merozoite proteins involved have not been completely elucidated (6). Attempts to unravel these components of NAI have led to the development of assays such as the enzyme-linked immunosorbent assay (ELISA) for the quantification of antibodies, but this does not always correlate with the functional capacity of the measured antibodies (7).

Antibodies may directly inhibit parasite growth (8) or, merozoite invasion of the red blood cells (9) or, facilitate complement fixation leading to parasite killing (10) or opsonize merozoites and infected erythrocytes (11), and blocking the sequestration of infected parasites (12). Evidence from several sero-epidemiological studies suggests that the most effective antibodies against malaria parasites are the cytophilic subclasses of IgG (i.e., IgG1 and IgG3) (13, 14). Consequently, mechanisms that involve antibody collaboration with effector cells such as the monocyte-mediated antibody-dependent cellular inhibition (ADCI) and opsonic phagocytosis (OP) (15–17) or the neutrophil-mediated antibody-dependent respiratory burst (ADRB) (18) have been developed and all been associated with protection against malaria in different study populations. Nonetheless, the antigenic targets of antibodies involved in these mechanisms have not always been consistent across different studies. Glutamate rich protein (GLURP) has been associated with OP (16). Also, antibodies to merozoite surface protein duffy binding-like (MSPDBL)1 and MSPDBL2 from the MSP3 family, have been shown to have strong opsonizing and growth inhibitory activities (19). Further, studies comparing the relationships between different malaria protective immune mechanisms in a single well-characterized cohort may reveal important dynamics that may be exploited in designing more effective malaria vaccines. For instance, antigenic targets that may be more specific to different mechanisms may be incorporated into a single multivalent vaccine to benefit from the multiple protective immune mechanisms against malaria. OP and ADCI were studied in the same cohort and were both independently associated with protection against malaria. There was however, no evidence of correlation between the two mechanisms, perhaps reflecting differences in the underlying pathways involved (16).

Here, we assessed the roles of OP and antibody dependent growth inhibition mechanisms, their inter-relationships and possible antigenic targets on the outcomes of *P. falciparum* infection in a longitudinal cohort study of children living in endemic communities of southern Ghana.

## Materials and methods

### Patient consent statement

Ethical approval was given by the institutional review board of the Noguchi Memorial Institute for Medical Research, University of Ghana, and the Ghana Health Service Ethics Committee, Ghana. Written informed consent was given by the guardians of the participants before enrollment into the study.

### Study site and participants

This longitudinal cohort study was undertaken in six adjoining communities across the La-Nkwantanang Madina Municipality and the Ga-East Municipality of the Greater-Accra Region in southern Ghana. The detailed study design, area, and population have been described elsewhere (20). Briefly, 973 children between the ages of 0.5 and 13 years were recruited into the study. About 3 ml venous (for children<2 years, only about 0.5ml of finger-prick blood was collected into a microtainer tubes) blood was collected into EDTA tubes at enrollment and children were monitored for malaria symptoms actively (weekly home visits) and passively over 50 weeks. Monthly parasitaemia surveillance was by finger-pricking and microscopy. At enrolment, febrile children with parasitaemia were excluded from the study during the initial screening. Children with baseline parasitaemia who become febrile during the follow-up period were added to the susceptible group. The monthly finger pricking was used for asymptomatic parasitaemia surveillance. If a child was parasite positive during the monthly finger pricking but does not develop fever, that child in considered asymptomatic.

### *P. falciparum* culture and merozoite purification

*P. falciparum* strain NF54 was maintained in culture in O+ human erythrocytes with RPMI-1640 medium (52400-025, Gibco™) supplemented with 1% L-glutamine (G7513-100, Sigma Aldrich), 50 µg/ml gentamycin (15750-045, Gibco™), 25 mM HEPES (Sigma Aldrich), 50 mg/L hypoxanthine, and 10% heat-inactivated type O+ human serum. Cultures were incubated at 37 °C with a gas mixture of 5% O_2_, 2% CO_2_, balanced with N_2_ (21).

Isolation of intact merozoites was performed as has been described elsewhere (22). Briefly, schizont stage parasites were harvested using magnetic-activated cell sorter (MACS) columns (Miltenyi Biotec, Bergisch Gladbach, Germany) and monitored for segmentation. Merozoites were harvested from the matured segmented schizonts using a 1.2-μm filter (Sartorius stedim biotech, Goettingen, Germany). Free merozoites were stained with 100 µg/ml ethidium bromide (Sigma Aldrich) for 30 minutes, washed twice by centrifuging at 1900xg (Kubota 5911) for 8 minutes at room temperature using RPMI-1640 supplemented with 10% Foetal Calf Serum (FCS), and counted using Count-Bright Absolute Counting Beads (Thermo fisher, Oregon, USA) following manufacturer’s protocol. Merozoites were resuspended at 8 x10^6^ in THP1 medium (RPMI-1640, supplemented with 1% L-glutamine, 0.5% pen strep,10% FBS Sigma Aldrich, and 5 x 10^−5^ mol/L 2-mercaptoethanol) and used in subsequent assays.

### THP1 cell line culture

THP1 cells were maintained in THP1 medium at 37^o^C in a humidified chamber with 5% CO_2_ gas. Cell numbers were maintained at 1×10^6^ cells/mL. Cells were counted, resuspended, and aliquoted at 1x10^4^ cells/150 µl/well for the opsonic phagocytosis assay.

### Opsonic phagocytosis assay

Opsonic phagocytosis assay was performed as described (16, 17). Briefly, 4x10^5^ merozoites were stained with 100 μg/ml ethidium bromide and opsonized with 50 μl heat-inactivated (HI) plasma (diluted 1:50) for 30 minutes at room temperature. THP1 cells (1x10^4^) were co-incubated with opsonized merozoites in a U bottom 96-well plate pre-coated with 150 μl/well FCS. The plate was incubated for about 40 minutes in a humidified incubator at 37^o^C supplemented with 5% CO_2_ gas. Phagocytosis was stopped by spinning the plate at 500xg in a pre-chilled centrifuge for 5 minutes at 4^o^C. The plate was washed twice with cold PBS supplemented with 2% BSA. Cells were fixed with 2% paraformaldehyde in PBS/2% BSA and counted using a Beckman Coulter cytometer (Cytomics FC500 MPL, Beckman Coulter Inc). Data were expressed as phagocytosis index, defined as the percentage of THP-1 cells with ingested merozoites relative to the THP1 cells with no merozoites.

### Growth inhibition assay

Growth inhibition assay was performed as previously described (23). Briefly, schizonts from NF54 parasite strain were purified using MACS columns (Miltenyi Biotec, Bergisch Gladbach, Germany) and put back into culture using the 96-well flat bottom plate in a volume of 50 μl/well with 50 μl/well test samples. In setting up the assay, parasitaemia was adjusted to 1% with a haematocrit of 2% and added to the plates. Test plasma, hyper-immune plasma (pooled plasma from 10 malaria-exposed Liberian adults used as a positive control), and naïve plasma (pooled plasma from 19 malaria-naïve Danish individuals used as the negative control) (15, 16) were added to their respective wells at 50 µl/well. All plasma was heat-inactivated (HI) before the assay. An assay control, ethylenediaminetetraacetic acid (EDTA) was added at 8 mM at 50 µl/well. For the monitoring and normal growth wells (control wells), 100 μl/well of parasite suspension (2% haematocrit, and 1% parasitaemia) was added. The assay plate was incubated in a humidified chamber at 37^o^C with a gas mixture of 5% O_2_, 2% CO_2_, balanced with N_2_. After approximately 12 hours of incubation, cultures were centrifuged at 500xg for 5 minutes in a pre-chilled centrifuge at 4^o^C to pellet the cells and washed with cold filtered 1xPBS. Cells were stained with 0.2x SYBR Green I nucleic acid stain (Sigma Aldrich) for 10 minutes and washed twice by spinning at 2500 rpm for 8 minutes. About 200 μl of cold PBS was added and the cells acquired with Accuri C6 Plus flow cytometer (Becton Dickinson), set to acquire 50,000 events. Growth inhibition was calculated and expressed as a percentage inhibition index using the formula: 100 − (100 × (test well/normal growth inhibition well). Test well is the well with test antibodies and parasitized RBCs, and control well represents the parasitized RBCs without antibodies.

The gating strategy was adopted from Muh et al. (23), where single erythrocytes were gated using FSC-A and FSC-H. From this, the infected and uninfected erythrocytes were gated with SYBR green 1 stained channel for the identification of early ring stages, which are the newly invaded parasites, from the late trophozoite and schizont stage.

To test the sensitivity of the assay to detect different samples at different concentrations, two sets of pooled plasma samples were diluted 10-fold from undiluted plasma to 1000x dilution and added to the culture in 96-well plates. Pool A was normal pooled plasma and pool B was HI pooled plasma. Both plasma pools demonstrated concentration (dilution)-dependent growth inhibitory effects on NF54 Plasmodium parasite (**Supplementary Figure 1**).

### Antibody measurements

The antigens, MSPDBL1, EBA140RIII-V, RALP-1, *Pf*Rh2A, *Pf*Rh2B, and RON4, used in this study have been described previously (24) and the ELISA protocol was described elsewhere (25). Briefly, 96-well flat-bottom plates (Nunc MaxiSorp, Denmark) were coated with antigens (100µl/well) at 0.5µg/ml in phosphate-buffered saline (PBS; pH 7.4) and incubated overnight at 4^o^C. Samples were added at 100µl/well diluted at 1:500 in sample diluent (PBS with 1% milk powder, 0.1% Tween 20, and 0.02% Na-azide) and incubated for 1hour at RT on a plate shaker set at 300rpm. Each plate had a standard reference curve obtained by a 3-fold serial dilution of pooled hyperimmune plasma. Also included on each plate were controls (Positive and Negative) added at 100µl/well. Total IgG was detected for an hour using 100µl/well of HRP conjugated goat anti-human IgG (H10307, Waltham, MA, USA) diluted at 1:2,000. The assay was developed with 3,3',5,5' Tetramethylbenzidine for 15minutes and optical density read at 450nm. The standard reference curve was used to convert OD values to antibody units using an Excel-based 4-parameter logistic curve-fitting application (ADAMSEL b040; Ed Remarque, 2009).

### Data normalization

All the three data sets of opsonic phagocytosis, growth inhibition, and IgG antibody data were normalized respectively, where the mean of the positive control of each assay output was averaged across replicates for each assay plate. The test sample on each plate was divided by the mean positive control of the plate and multiplied by the total mean positive control for all the plates to obtain the adjusted value for the sample using the formula:


$$\left(\text{Sample}/\text{Plate Positive control}\right)\times \text{Mean positive control for all plates}$$

### Outcome and participant categorization

Participants with *P. falciparum* parasites detected by microscopy at any time during the 50 weeks were categorized as the exposed group and all those without parasites throughout the observation period were considered as unexposed. Though children above 5 years have been observed to have sub-microscopic infection (20), the gold standard, microscopy, which is mostly used in diagnosing malaria was used in the categorization of the study participants The unexposed were excluded from this analysis. The exposed group was further categorized based on whether they had febrile malaria (defined as fever, measured axillary temperature of ≥ 37.5^o^C, or reported, and any other symptoms of clinical malaria such as chills and malaise) at any time during the 50 week follow up, or were asymptomatic throughout the same period. Participants with parasites at least one-time point but no febrile symptoms were considered protected, and those with at least one episode of febrile malaria during the follow-up period were considered susceptible.

### Statistical analysis

All analyses were done separately for the age groups (< 5 years old and > 5 years old). Baseline characteristics were summarized using means and standard deviations for continuous variables and counts and frequencies for categorical variables. Associations between OP and the odds of protection against febrile malaria were assessed using multiple logistic regression adjusted for age. These analyses were repeated for growth inhibition.

To generate antibody breadth score, antibody levels were grouped based on quartiles and assigned 0 for the lowest quartile, 1 for the second quartile, 2 for the third quartile, and 3 for the highest group. The scores for each individual were summed up for the 6 antigens with 18 being the highest breadth and the least being zero (0). An association of breadth with protection was assessed using logistic regression analysis adjusting for age. The associations between breadth score and the immune mechanisms were assessed by simple linear regressions separately.

A multiple linear regression analysis adjusted for age was used to determine the correlation between the immune mechanisms (OP and growth inhibition) and antigen-specific total IgG antibody levels. Similarly, multiple linear regression was also used to explore the correlation between OP and growth inhibition. The level of statistical significance was set at 5%. All analyses were performed using R (26).

## Results

### Study population characteristics

The study enrolled a total of 973 children (aged 0.5-13 years old) between January 2016 and January 2017. A total of 848 children completed the 50-week longitudinal follow up period. At the end of the study, 238 children were infected with *P. falciparum* parasite at least once during the follow-up period as confirmed by microscopy (exposed group) (**Supplementary Table 1**). There were fewer children under 5 years (33.2%) in the exposed group compared to those above 5 years (p<0.0001). The proportions of other covariates were similar between the two groups. All subsequent analyses were done using only the exposed group. (**Table 1**). The distribution of the covariates, sex, bed net use, haemoglobin, blood group and baseline parasitaemia between the two age groups is shown in **Table 1**.

**Table 1:** Baseline characteristics .

Variable	Below 5 yrs (n=79)	Above 5 yrs (n=159)
Sex		
Male	42 (53.2)	90 (56.6)
Female	37 (46.8)	69 (43.4)
Bed net		
No	55 (69.6)	93 (58.5)
Yes	24 (30.4)	66 (41.5)
Blood Group		
O	32 (48.5)	64 (51.2)
A	16 (24.2)	29 (23.2)
AB	3 (4.5)	6 (4.8)
B	15 (22.7)	26 (20.8)
Missing	13	34
Hb, mean (sd)	10.4 (1.7)	11.4 (1.3)
Missing	19	32
Baseline Parasitaemia		
Negative	61	110
Positive	18	49

IQR is the interquartile range.

Data are reported as n (%) unless otherwise specified.

Sd in the standard deviation.

### Relationship between OP and growth inhibition activity and baseline parasitaemia

**Figure 1:**
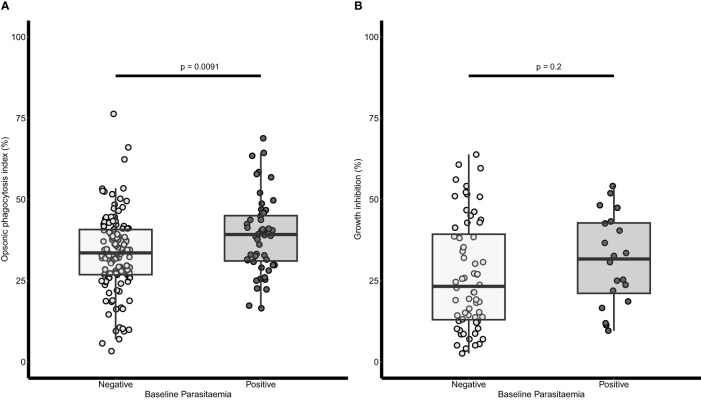
Relationship between baseline parasitaemia and opsonic phagocytosis (OP) and growth inhibition respectively. **(A)** A dot plot of OP and baseline parasitaemia (n=238). **(B)** A dot plot of growth inhibition activity and baseline parasitaemia (n=84). Parasitaemia was defined as any parasite count by microscopy examination of blood slides. The lighter dots and boxes are for children without baseline parasitaemia and the darker dots and boxes are for those with baseline parasitaemia. The horizontal lines within the boxes are the median of the distribution, the upper and lower borders of the box denote the upper and lower quartiles respectively. The upper and the lower whiskers indicate the maximum and minimum OP values respectively. P values were derived from the Mann Whitney U test.

The OP and growth inhibition activities of baseline plasma samples were compared between children who had baseline parasitaemia and those without baseline parasitaemia separately. Children with baseline parasitaemia had a significantly higher OP activity (Mann Whitney U test, p = 0.0091) than those without baseline parasitaemia (**Figure 1A**). Similarly, the growth inhibition activity appeared higher in children with baseline parasitaemia than those without baseline parasitaemia, but this was not statistically significant (**Figure 1B**).

### OP and growth inhibition activity increased with age and was higher in children protected against febrile malaria

**Figure 2:**
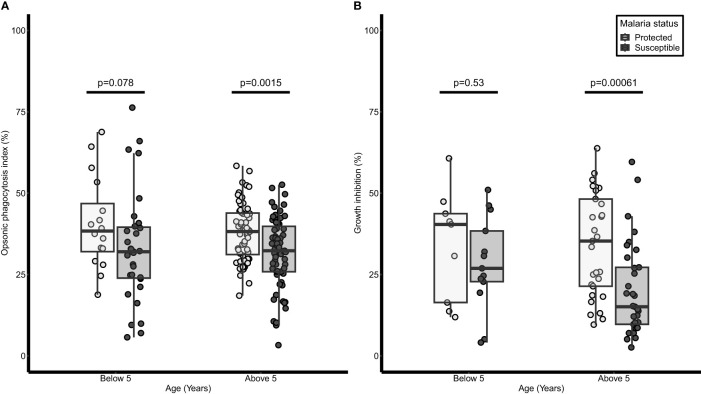
Opsonic phagocytosis and growth inhibition activities are higher in older children and are associated with protection against malaria. A dot and box plot of: **(A)** opsonic phagocytosis activity (n=238), **(B)** growth inhibition activity (n=84); for children below 5 years and those above 5 years we were protected from or susceptible to malaria. The lighter dots and box represent protected children while the darker dots and box represent susceptible children. The horizontal line within each box denotes the median of the distribution, the upper and lower borders of the box denote the upper and lower quartiles respectively. The upper and the lower whiskers indicate the maximum and minimum OP values respectively. P values were derived from the Mann Whitney U test.

In a logistic regression analysis, age was associated with protection from febrile malaria [odds ratio (OR) = 0.85, 95% CI = 0.78-0.92; p<0.001]. Similarly, OP activity was associated with protection from febrile malaria [adjusted (aOR)= 0.16; 95%CI= 0.05 - 0.50, p = 0.002]. When the children were grouped into two based on age, there was no difference in the OP activity between protected and susceptible children (p=0.078) in the younger age group (Below 5 years). However, for those above five years, OP activity was higher in the protected children (33.11 ± 1.15 %) compared to the susceptible children (31.76 ± 1.31 %, p=0.0015) (**Figure 2A**).

There were 84 samples (38 protected, and 46 susceptible) available for analysis in the growth inhibition assay due to insufficient plasma samples. Plasma from protected children above 5 years had higher growth inhibition activity (34.19 ± 2.95 %) compared to susceptible children (20.14 ±2.49 %, p= 0.00061) in the same age group while no significant difference was observed in those below 5 years (p=0.53) (**Figure 2B**). Both age groups were then combined and after adjusting for age, high inhibition activity was associated with protection from febrile malaria (aOR=0.15; 95% CI = 0.04-0.47; p = 0.001).

### Relationship between OP activity, growth inhibition, and naturally acquired antibodies

Antigen-specific IgG antibody levels to six (6) malarial antigens were measured by ELISA to determine their relationship with OP and growth inhibition, and their association with protection from febrile malaria, respectively. Total IgG levels to MSP1DBL1 (p=0.001) and RON4 (p=0.015) were associated with protection from febrile malaria (**Table 2**). The linear relationship between OP and total IgG levels against the 6 merozoite antigens were explored. Total IgG levels to MSP1DBL1, and RON4 significantly increased with OP activity (p=0.003 in both antigens). Similarly, total IgG levels to *Pf*Rh2a (p=0.04), and RON4 (p=0.004) significantly increased with growth inhibition activity (**Table 3**). Antibodies to RON4 significantly increased with both OP and growth inhibition suggesting RON4 may be a target for both mechanisms (**Table 3**). We then investigated the effect of age on the linear relationship between total IgG levels and both OP and growth inhibition. We found that children above five (5) years of age had significantly increased levels of RON4 antibodies with increasing levels of both OP activity and growth inhibition respectively. On the other hand, MSP1DBL1 antibody levels was only significantly increased with OP activity but not growth inhibition. (**Table 4**).

**Table 2:** Association between total IgG levels and protection against febrile malaria.

Antigen	Total IgG and protection against malaria		
	aOR	95 % CI	p-value
EBA140RIII-V	0.88	0.62 to 1.25	0.47
MSPDBL1	0.59	0.42 to 0.82	**0.001**
PfRh2a	0.83	0.53 to 1.29	0.40
PfRh2b	0.97	0.75 to 1.26	0.82
RALP1	0.74	0.50 to 1.10	0.13
RON4	0.75	0.59 to 0.94	**0.015**

Odds ratios (aOR), 95% confidence intervals (95%CI) and p-values for each antigen were obtained with multiple logistic regression models adjusting for age. Significant p-values are in bold font.

**Table 3:** Association between total IgG responses and opsonic phagocytosis and growth inhibition respectively.

Total IgG	Opsonic Phagocytosis			Growth Inhibition		
Antigen	β -Coefficient	95 % CI	p-value	β -Coefficient	95 % CI	p-value
EBA140RIII-V	1.28	-0.83 to 3.38	0.23	4.06	-0.93 to 9.04	0.11
MSPDBL1	2.67	0.93 to 4.46	**0.003**	2.60	-1.03 to 6.24	0.16
PfRh2a	0.64	-2.19 to 3.47	0.65	5.97	0.37 to 11.58	**0.04**
PfRh2b	1.43	-0.23 to 3.08	0.09	2.57	-0.63 to 5.77	0.12
RALP1	1.51	-0.72 to 3.74	0.18	3.16	-1.81 to 8.16	0.21
RON4	1.92	0.65 to 3.20	**0.003**	4.25	1.41 to 7.09	**0.004**

β coefficients, 95% confidence intervals (95%CI) and p-values for each antigen were obtained with linear regression models adjusting for age. Significant p-values are in bold font.

**Table 4:** Association between total IgG levels and functional antibodies in opsonic phagocytosis and growth inhibition assays.

Total IgG	Opsonic Phagocytosis			Growth Inhibition		
Antigen	β -Coefficient	95 % CI	p-value	β -Coefficient	95 % CI	p-value
Below 5 years						
EBA140RIII-V	3.89	-1.33 to 9.11	0.15	5.66	-3.26 to 14.56	0.23
MSPDBL1	3.72	-1.11 to 8.54	0.14	4.86	-3.25 to 12.96	0.25
PfRh2a	0.55	-6.04 to 7.13	0.87	9.41	-7.53 to 26.36	0.29
PfRh2b	4.15	-0.09 to 8.39	0.06	3.56	-2.81 to 9.93	0.29
RALP1	1.27	-3.47 to 6.01	0.60	7.97	-5.94 to 21.75	0.28
RON4	2.72	-0.21 to 5.65	0.07	3.10	-2.55 to 8.75	0.29
Above 5 years						
EBA140RIII-V	0.18	-1.90 to 2.27	0.86	3.10	-2.55 to 8.75	0.26
MSPDBL1	2.34	0.66 to 4.02	**0.007**	2.13	-1.98 to 6.24	0.31
PfRh2a	0.70	-2.22 to 3.61	0.64	5.59	-0.45 to 11.63	0.07
PfRh2b	0.44	-1.17 to 2.06	0.59	2.29	-1.44 to 6.01	0.23
RALP1	1.69	-0.74 to 4.12	0.17	2.56	-2.88 to 7.99	0.36
RON4	1.49	0.17 to 2.82	**0.029**	4.62	1.31 to 7.93	**0.008**

β coefficients, 95% confidence intervals (95%CI) and p-values for each antigen were obtained with linear regression models for each age group. Significant p-values are in bold font.

### Relationship between antibody breadth, OP and growth inhibition of merozoites

**Figure 3:**
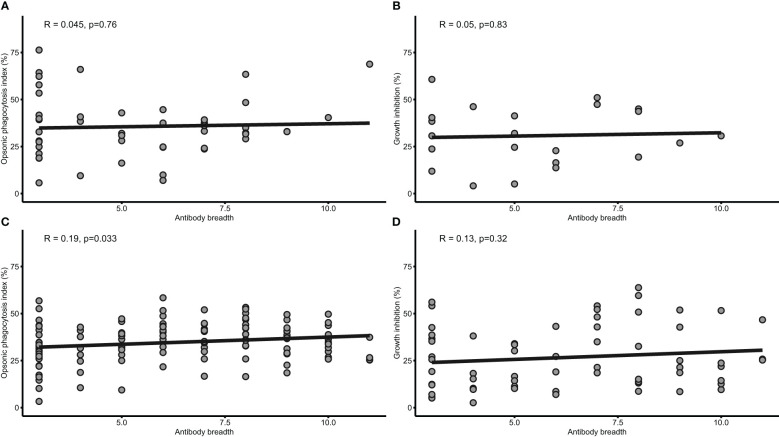
Antibody breadth is associated with both opsonic phagocytosis and growth inhibition. Scatterplots with linear regression lines are shown for the relationship between IgG antibody breadth and OP (n=79) **(A)**, and growth inhibition (n=22) **(B)** in children under five years, and OP **(C)** and grown inhibition **(D)** in children above 5 years. The shaded area around the regression line is the 95% Confidence Interval.

Both OP and growth inhibition are mediated by antibodies and have shown associations with protection against febrile malaria independently in this cohort. Hence, we investigated whether a relationship exist between the two antibody-mediated mechanisms using a linear model adjusting for age. There was no significant correlation (β= 0.13; 95% CI= -0.04-0.30; p=0.14) between the OP and growth inhibition activities. The breadth of total IgG responses was assessed for associations with age, malaria and the two mechanisms. Higher total IgG breadth was associated with age (β= 0.26; 95%CI=0.10-0.41; p=0.0012) and protection (aOR= 0.92; 95%CI= 0.86-0.99; p= 0.022) against febrile malaria. Also, total IgG breadth positively correlated with only opsonic phagocytosis (p=0.033) in those above five years, but not growth inhibition (p<0.05) (**Figure 3**).

## Discussion

In this study, we found both merozoite OP and growth inhibition activity of plasma samples obtained from children at baseline to be associated with protection against malaria in the ensuing transmission season. In addition, the breadth of total IgG responses against a panel of 6 merozoite antigens was associated with protection and increased with both OP and growth inhibition activities. There was no evidence of correlation between OP and growth inhibition, suggesting these could be contributing independently to the protection against malaria observed in the study population. Notably, RON4 specific antibody levels was significantly associated with both mechanisms.

The mechanisms and targets of antibody-mediated immunity to malaria are still under investigation and some studies have found OP (16, 17), ADRB (18), ADCI (15), GIA (27) and complement fixation (4) to be associated with protection from malaria. While these mechanisms appear to be individually associated with protection, their correlation with each other is not well established (16). In the current study, antibody-mediated OP and growth inhibition were performed in the same cohort and found to both protect against febrile malaria but did not correlate with each other though a weak positive association between the two was observed. It is possible that these two mechanisms target different parasite proteins and are therefore mediated by different antibodies. Also, the lower number of samples used in the growth inhibition assay might have contributed to the lack of significance. Nonetheless, the combined effects of both mechanisms and others not studied here may synergize in mediating protection against malaria. This may partly explain our observation that children whose plasma gave lower levels of both OP and growth inhibition activities were at a higher risk of malaria. We found IgG levels against MSPDBL1 and PfRh2a to correlate with OP and growth inhibition respectively while antibodies to RON4 correlated with both mechanisms. It is conceivable that antigenic targets such as RON4 shared by different mechanisms may be crucial in designing vaccines that could trigger both and possibly result in better efficacy.

Of the 6 recombinant antigens tested, higher antibody levels to MSPDBL1 and RON4 correlated with reduced risk of febrile malaria. Anti-MSPDBL1 antibodies have been found in other studies to confer protection by inhibition of parasite growth and also by opsonization of merozoites (19, 28). PfRh2a and RON4 are both rhoptry proteins and involved in erythrocyte invasion. RON4 forms complexes with RON2 which interact with AMA1 and other rhoptry neck proteins and participate in the formation of tight junction leading to erythrocyte invasion (29, 30). However, in the sporozoite stage, RON4 may act independently to aid invasion of hepatocytes (31). These agree with our observation that antibodies to PfRh2a and RON4 are associated with merozoite growth inhibition in the current study. In other studies, antibodies to RON4 and other rhoptry neck proteins were associated with protection in Ugandan and Papua New Guinean populations (32, 33) which is consistent with our current findings. Also, a study in Ghana using different cohorts found antibodies to RON4 to be protective from febrile malaria (5). A recent study has also found a complex involving SURFIN4.2, RON4 and GLURP to be important in the invasion of erythrocyte by merozoites (34). This may explain the positive correlation observed between RON4 antibodies and both OP and growth inhibition respectively, and thus the association with protection against malaria in these different studies which supports its suitability as a vaccine candidate. Notwithstanding, further studies using affinity purified antibodies against RON4, MSPDBL1 and PfRh2a will further consolidate their role in these malaria protective immune mechanisms.

There may be several other malaria antigen-specific antibodies in the plasma samples used that may be involved in the OP and growth inhibition mechanisms which have not been measured here which is a limitation. Another possible limitation is the impact of these mechanisms on different strains of parasites circulating in the population which we have not assessed here. However, we believe the plasma used in the assays have wider antibody breadth since a study in Papua New Guinea found similar OP activity in both field and laboratory strains indicating that opsonization of merozoites is strain transcending (35).

In conclusion, the study found both opsonic phagocytosis and growth inhibition mechanisms to independently mediate protection from febrile malaria in this cohort of Ghanaian children. The study also shows the importance of total IgG responses to RON4 in both OP and growth inhibition mechanisms suggesting that vaccines based on this antigen may trigger both and perhaps contribute to enhanced efficacy.

## Data availability statement

The raw data supporting the conclusions of this article will be made available by the authors, without undue reservation.

## Ethics statement

The studies involving human participants were reviewed and approved by Institutional Review Board of the Noguchi Memorial Institute for Medical Research, University of Ghana. Written informed consent to participate in this study was provided by the participants’ legal guardian/next of kin.

## Author contributions

Conceived and designed the experiments: EK-B, DD, MT, KAK and BA. Performed the experiments: EK-B, RT, EO-Y, and SS. Analyzed the data: EK-B, BA, TG, and SF. Contributed reagents/materials/analysis tools: SS, TG and MT. Wrote the manuscript: EKB, KAK, FKNA, BA, and MT. All authors contributed to the article and approved the submitted version.

## Supplementary material

Supplementary Figure 1 Growth inhibition is concentration dependent. Normal plasma (Dark bars) and Heat inactivated plasma (Grey bars) were serially diluted to test the inhibition growth assay protocol. The pool included plasma from 10 hyperimmune individuals.
