# Supplementary figures and images for: High opsonic phagocytosis activity and growth inhibition of merozoites are associated with RON4 antibody levels and protect against febrile malaria in Ghanaian children
Source: Front Immunol. 2023 May 1;14:1161301. doi: 10.3389/fimmu.2023.1161301 (PMC10183564; doi:10.3389/fimmu.2023.1161301)

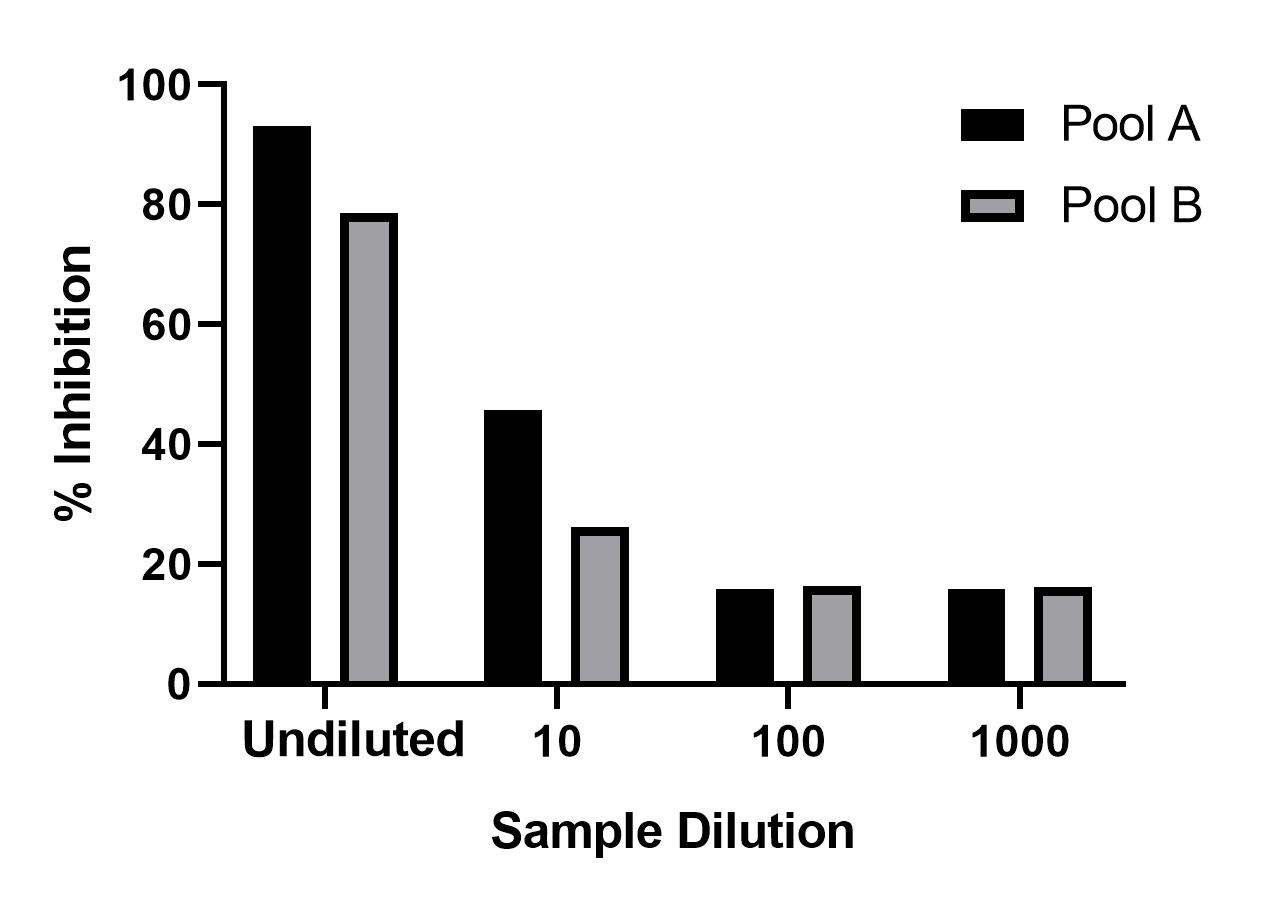

Supplement: Supplementary Figure 1 — Growth inhibition is concentration dependent. Normal plasma (Dark bars) and Heat inactivated plasma (Grey bars) were serially diluted to test the inhibition growth assay protocol. The pool included plasma from 10 hyperimmune individuals. [file Image_1.jpeg]
