# Supplementary material for: High opsonic phagocytosis activity and growth inhibition of merozoites are associated with RON4 antibody levels and protect against febrile malaria in Ghanaian children
Source: Front Immunol. 2023 May 1;14:1161301. doi: 10.3389/fimmu.2023.1161301 (PMC10183564; doi:10.3389/fimmu.2023.1161301)
Supplement: Supplementary file 2 [file Table_1.docx]

**Supplementary Table 1: Association between variables and exposure to *P. falciparum* parasites**

| **Variable** | **Unexposed (n=735)** | **Exposed**  **(n=238)** | **OR (95% CI)** | **p-value** |
| --- | --- | --- | --- | --- |
| Age |  |  |  |  |
| Below 5yrs | 362(49.3) | 79(33.2) | Ref. |  |
| Above 5 yrs | 373(50.7) | 159(66.8) | 0.51 (0.38-0.70) | <1e-04 |
| Sex |  |  |  |  |
| Female | 349(47.5) | 106(44.5) | Ref. |  |
| Male | 386(52.5) | 132(55.5) | 0.89 (0.66-1.19) | 0.43 |
| Bed net |  |  |  |  |
| No | 449(61.1) | 148(62.2) | Ref. |  |
| Yes | 286(38.9) | 90(37.8) | 1.05 (0.78-1.42) | 0.76 |
| Blood Group |  |  |  |  |
| A | 134(23.6) | 45(23.6) | Ref. |  |
| B | 129(22.7) | 41(21.5) | 1.06 (0.65-1.72) | 0.82 |
| O | 278(48.9) | 96(50.3) | 0.97 (0.65-1.46) | 0.89 |
| AB | 27(4.8) | 9(4.7) | 1.01 (0.44-2.30) | 0.98 |
| Missing | 167 | 47 | 214 |  |
| Hb mean (Sd) | 11.2 (1.6) | 11.1 (1.5) | 1.08 (0.97-1.19) | 0.15 |
| Missing | 159 | 51 | 210 |  |

OR and 95%CI were generated from a simple logistic regression

Sd is the standard deviation

Data are reported as n (%) unless otherwise specified
